# Gaseous Heptanethiol Removal by a Fe^3+^-Phenanthroline–Kaolinite Hybrid Material

**DOI:** 10.1021/acsomega.1c04145

**Published:** 2021-11-23

**Authors:** Fabrizio Bernini, Elena Castellini, Maria Franca Brigatti, Beatrice Bighi, Marco Borsari, Daniele Malferrari

**Affiliations:** Department of Chemical and Geological Sciences, University of Modena and Reggio Emilia, Via Campi 103, I-41125 Modena, Italy

## Abstract

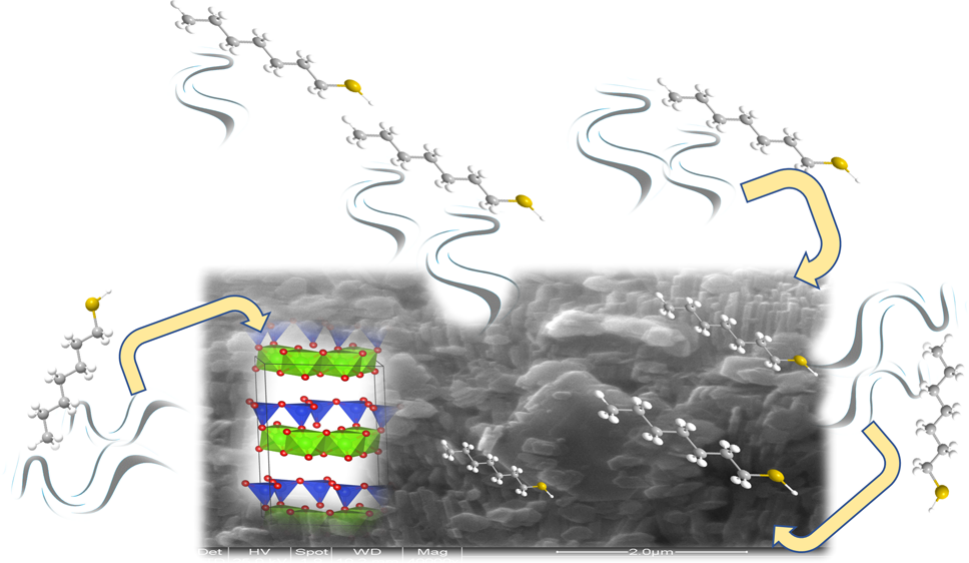

Kaolinite functionalized by the μ-oxo Fe^3+^-phenanthroline complex (Fe^+3^Phen) was selected to test its ability to efficiently remove and store gaseous heptanethiol (HPT). Spectroscopic techniques, elemental analysis, and thermal analysis coupled with evolved gas mass spectrometry were employed to characterize the material before and after the exposure to the gas and to define the adsorption process. The amount of HPT trapped by the functionalized kaolinite after 60 days is 0.10940 moles per 100 g of kaolinite which, considering the amount of adsorbed Fe^+3^Phen (0.00114 moles per 100 g of kaolinite), means a thiol/Fe^3+^Phen molar ratio of about 100:1, a value much higher than those found in the past for Fe^+3^Phen functionalized montmorillonite and sepiolite. In addition, the process was found to be efficient also beyond 60 days. This significant removal of the smelly gas was explained by considering a continuous catalytic activity of Fe^3+^ toward the oxidation of thiol to disulfide.

## 1 Introduction

Hybrid clay materials (HCMs) produced through the functionalization of layer silicates with different organic and inorganic molecules are one of the main focus areas for applicative and technology-oriented clay research. Usually, these materials are grouped based on the type of host (i.e., the layered mineral) and guest molecule involved in the interaction. Three main types of functionalized layer silicates can be identified, namely, (i) organoclays, which are layered silicates (typically smectites) bound to an organic molecule (or to an organo-metallic complex) that imparts peculiar properties to the modified minerals;^1−7^ (ii) pillared clays, which are layered silicates intercalated with small organic or inorganic complexes (i.e., “pillars”), partially filling the interlayer space;^8−13^ and (iii) clay mineral-nanocomposites, which are fine-particulate materials, heterogeneous at the nanoscale level, made at least from one clay mineral and from other materials such as polymers, pharmaceuticals, inorganic molecules, or carbon.^14−23^

2:1 layer silicates such as smectites are excellent candidates for the formation of HCMs; in fact, because of their high cation exchange capacity and swelling behavior, they can intercalate even through fast one-step reaction cations or polar molecules that give peculiar properties to the resulting structure.^14,15,24−27^ On the other hand, the intercalation of cations and molecules in 1:1 layer silicates such as kaolinite, although it is possible,^28−34^ is more difficult as the interactions are usually limited to exposed edges and sheets (adsorption), leaving the layer structure almost unchanged. In fact, unlike in smectite, kaolinite layers are bound by reactive hydrogen bonds which could not be easily broken;^35^ consequently, even if the basal spacing of kaolinite can be extended to values higher than the original 0.71 nm, it is very difficult to induce swelling through a fast one-step reaction. Furthermore, smectite is able to capture a considerably larger number of cations/molecules than kaolinite, and it is the preferred choice in many applications such as, for example, pollutant trapping. Because of this background, kaolinite has been so far less commonly used than other clay minerals for the preparation of organic–inorganic HCMs.

Past research studies demonstrated that HCMs can be obtained by reacting kaolinite, montmorillonite, and sepiolite (a modulated 2:1 layer silicate) rich clays with a solution containing the μ-oxo Fe^3+^-phenanthroline 1:1 complex [(OH_2_)_3_(Phen)FeOFe(Phen)(OH_2_)_3_] (Fe^+3^Phen hereafter).^36−39^ Later, Fe^+3^Phen-functionalized montmorillonite was successfully used to selectively capture, also at very low partial pressure of the gas, volatile organic sulfur derivatives,^39,40^ hydrogen sulfide,^41^ naphthalene, and Cl-naphtalene.^42^ Besides, HCMs obtained adsorbing Fe^+3^Phen on sepiolite demonstrated a better trapping ability toward thiols than montmorillonite,^39^ even if the efficiency of this catalytic process was significantly affected by the structural features of this modulated 2:1 layer silicate. Similarly, high trapping ability toward hydrogen sulfide and ammonia gas was observed also for montmorillonite-based HCMs obtained by intercalation of the Cu^2+^-phenanthroline complex.^43,44^ Furthermore, the Cu^2+^-phenanthroline complexed montmorillonite, after being used for trapping volatile thiol, can be successfully reused to capture aromatic halobenzenes from the gas phase.^45^

The use of natural or modified kaolin (a kaolinite rich clay) as a possible trap for sulfur-bearing gas was rarely considered in the past. The first promising results have been obtained for chemically and thermally treated kaolin.^46,47^ More specifically, these treatments improve the gas-trapping ability of the clay as the heating originates from amorphous silica, whereas the chemical treatment (acid or caustic) removes the structural ions and promotes the formation of Si–OH groups responsible for an enhanced gas adsorption. Later,^48^ the H_2_S adsorption capacity of natural kaolin was found to be dependent on the gas flow rate and temperature, but with a performance very low compared to other synthetic materials such as, for example, synthetic Zn-activated zeolite,^49^ so much that authors rightly concluded that kaolin could be an effective sorbent for H_2_S, but only if properly modified to increase its performance. In this direction, encouraging results have been obtained by functionalizing the kaolinitic clay with polyethyleneimine to prepare a material able to trap representative aldehyde, carboxylic acid, and disulfide volatile organic compounds.^50^ Nevertheless, to our knowledge, no further research was subsequently carried out using kaolinite to trap gaseous compounds, probably because of its “known lower performance” compared to other clay minerals and synthetic materials.

This study addresses the application of a Fe^3+^Phen kaolinite-based material (Kt-Fe^3+^Phen) as an effective and high performing trap for volatile heptanethiol (HPT). Because kaolinite binds an almost negligible amount of Fe^3+^Phen (i.e., the active adsorption center) with respect to montmorillonite and sepiolite,^36,39^ its trapping ability toward volatile thiol was expected to be lower, as well in absolute terms. However, the HPT adsorption normalized to one mole of Fe^3+^Phen is several times higher in Kt-Fe^3+^Phen than in Fe^3+^Phen-functionalized montmorillonite and sepiolite, and the results of this research will explain the reasons.

## 2 Materials and Methods

### 2.1 Preparation of Kt-Fe^3+^Phen

The kaolinite (Kt) used in this work is the reference clay material kaolinite KGa-1b from the Clay Minerals Society (The Clay Minerals Society, Source Clays Repository, University of Missouri, Columbia, MO). All chemical products used are of analytical grade (purity >99%) supplied by Carlo Erba (acetic acid, Fe_2_(SO_4_)_3_·8H_2_O and NaOH pellets) and by Sigma-Aldrich (1,10-phenanthroline and 1-HPT). The method to prepare the Fe^3+^Phen solution as well as the adsorption mechanisms of the organometallic complex on Kt was discussed in detail in the past research.^36^ Following the same procedure, fresh aliquots of Kt-Fe^3+^Phen were obtained under Fe^3+^Phen adsorption under equilibrium conditions. In short, 20 mg of Kt were suspended in 4 mL of a 0.15 mM Fe^3+^Phen solution and shaken at 250 rpm in an orbital incubator (Stuard Scientific Orbital Incubator SI50) at 20 °C for 30 min. The solid phase was then separated from solution, washed several times with acetate buffer and then with Millipore water, and finally air-dried at 20 °C.

### 2.2 Gas-Trapping Test

The gas-trapping ability of Kt-Fe^3+^Phen was tested for HPT vapor. HPT immobilization was achieved at 20 °C in a closed glass box. Different glass containers were prepared to host 50 mg of Kt-Fe^3+^Phen and uniformly spread on the bottom of a Petri dish (diameter = 50 mm), together with a beaker containing 3 mL of HPT. As already experimented with other HCMs,^39,40^ this volume is sufficient to ensure vapor saturation even in the case of a high degree of uptake. After fixed times of exposure, ranging between 12 h and 60 days, the samples were removed from the Petri dishes, air-dried for 1 h, and stored in plastic sealed containers. The sample exposed for 60 days is hereafter identified as Kt-FePhen-HPT-60d.

### 2.3 Analytical Methods

As in the past research aimed at studying the uptake and retention of gaseous compounds by the HCM mentioned in the Section 1, also the characterization of the Kt-Fe^3+^Phen before and after exposure to HPT was carried out through a multianalytical approach which encompasses: (i) elemental analyses to quantify carbon, nitrogen, and sulfur and thus calculate the amount of adsorbed complex and trapped thiol; (ii) thermogravimetric analysis (TGA) coupled with evolved gas mass spectrometry (MSEGA) to measure the temperature at which the complex and HPT are released by heating; and (iii) UV–vis and infrared (IR) spectroscopy methods to detect the mechanism of HPT uptake. Details of instruments and applied experimental conditions are reported in the on-line Supporting Information.

## 3 Results

### 3.1 Chemical Analyses

The amounts of nitrogen (0.063 wt %), carbon (0.361 wt %), and sulfur (0.036 wt %) measured through elemental analysis of Kt-Fe^3+^Phen are in good agreement with those already found by adsorption isotherms in the past.^36^ The occurrence of sulfur in the not-exposed sample is ascribed to the adsorption of SO_4_^2–^ counterions from solution, as the Fe^3+^Phen was prepared using phenanthroline and Fe_2_(SO_4_)_3_·8H_2_O. The amount of adsorbed complex, calculated from the nitrogen concentration, is 0.00114 Fe^3+^Phen moles per 100 g of Kt, and it is consistent with the same datum obtained from carbon (i.e., the experimental and theoretical N/C molar ratios are nearly the same).

**Figure 1:**
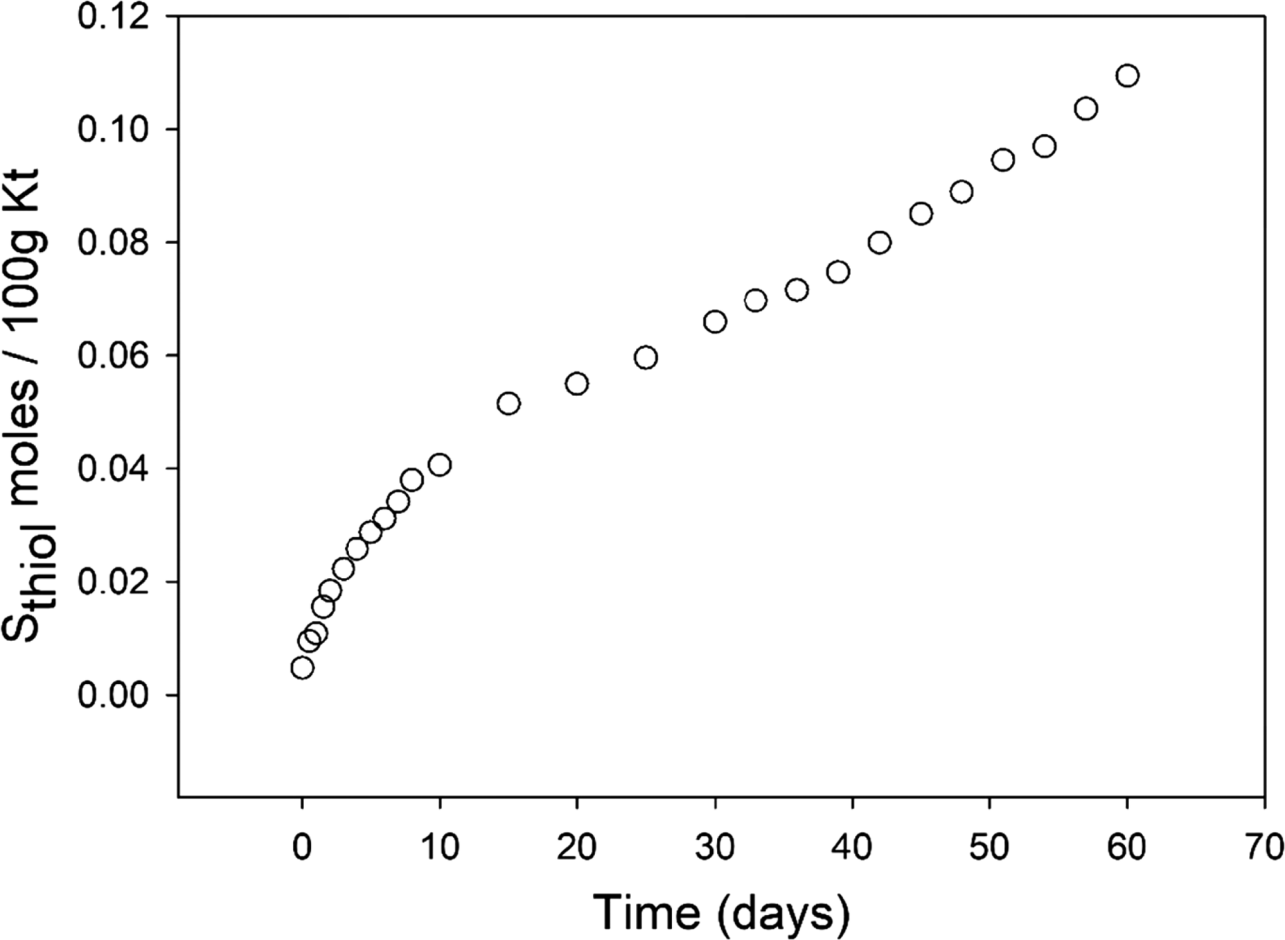
HPT adsorption kinetics. Moles of sulfur from thiol (S_thiol_) referred to 100 g of Kt measured in Kt-Fe^3+^Phen samples exposed to HPT for different times. Standard deviation is inside the dimension of the symbol.

The adsorption kinetics of HPT, plotted as moles of sulfur, is presented in Figure 1. Each point of the kinetic curve represents the difference between total sulfur and that already present in Kt-Fe^3+^Phen (i.e., 0.036 wt %, corresponding to 0.00115 moles per 100 g of Kt) which is due to the coadsorbed sulfate anions. Already after 1 day of exposure, the amount of sulfur is much higher (about 0.004 moles of sulfur per 100 g of Kt) than that in the not-exposed sample, and it progressively increases without reaching saturation. In fact, the kinetic curve clearly indicated that, even after 60 days of exposure, when the amount of trapped HPT is of 0.10940 moles per 100 g of Kt, the adsorption is far from reaching a steady state.

### 3.2 Thermal Analyses Coupled with Evolved Gas Mass Spectrometry

**Figure 2:**
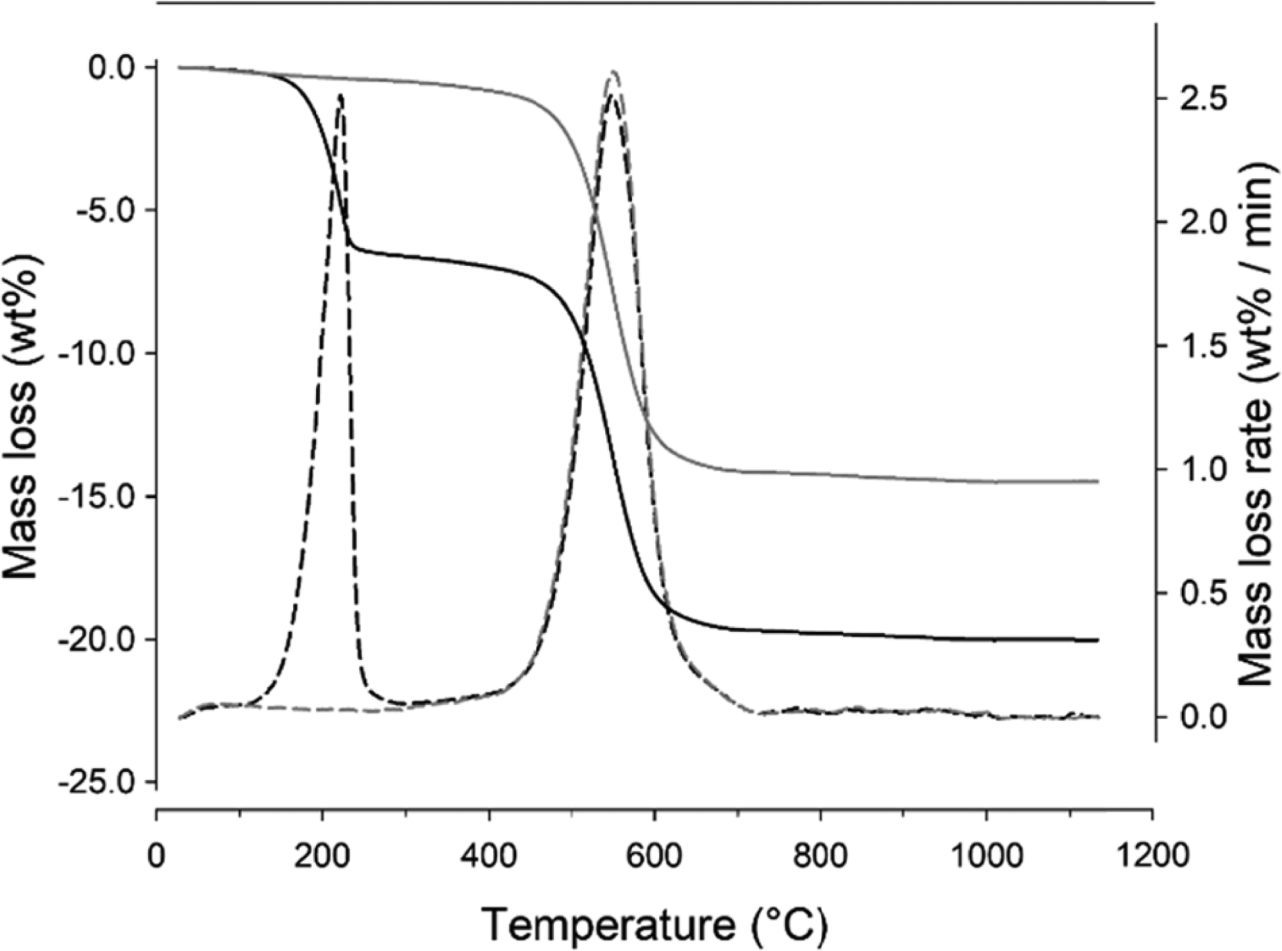
Thermal analyses. TGA (solid lines) and DTG curves (dashed lines) for samples Kt-Fe^3+^Phen (gray lines) and Kt-FePhen-HPT-60d (black lines).

**Figure 3:**
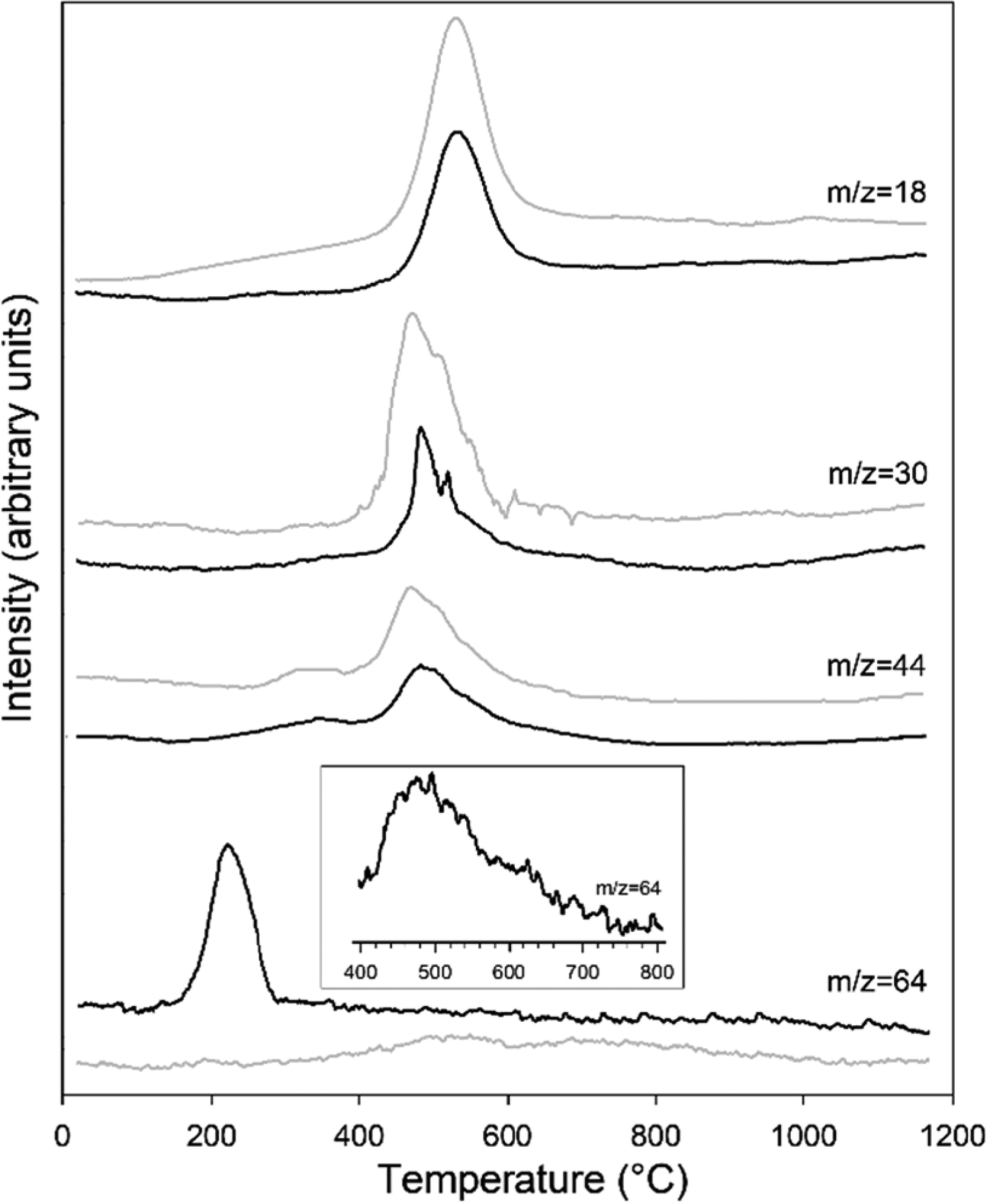
Evolved gas mass spectrometry. MSEGA curves detecting the release of H_2_O (*m*/*z* = 18), NO (*m*/*z* = 30), CO_2_ (*m*/*z* = 44), and SO_2_ (*m*/*z* = 64) for samples Kt-Fe^3+^Phen (gray lines) and Kt-FePhen-HPT-60d (black lines). In the rectangle is reported a magnification of the thermal range 400–800 °C for sample Kt-FePhen-HPT-60d.

Figure 2 compares the TGA curves of Kt-Fe^3+^Phen and Kt-FePhen-HPT-60d. In the derivative thermogravimetry (DTG) curve (i.e., the first derivative of TGA) a thermal reaction with maximum at 220 °C is present only in the sample exposed to HPT. As shown by MSEGA curves (Figure 3), it is related to the emission of SO_2_ (*m*/*z* = 64) and involves a mass loss of 6.6 wt %, corresponding to 0.104 moles of sulfur per 100 g of Kt, consistently with elemental analyses. The formation of SO_2_ as the thermal decomposition product is due to the occurrence in the oven of oxygen (the He gas flow does not saturate the atmosphere). Although the material exposed to HPT is highly hydrophobic, it cannot be entirely excluded that a small change in mass is due to the presence of physio-adsorbed water as indicated by the progressive increase of the signal of water (*m*/*z* = 18, Figure 3). A minor thermal effect, evidenced only by MSEGA curves, occurs at about 320 °C, and it is attributed to the (partial) thermal decomposition of the Fe^3+^Phen complex with the release of CO_2_, (*m*/*z* = 44). The same reaction was observed also in Fe^3+^Phen-functionalized montmorillonite and sepiolite,^36,39^ thus suggesting that this thermal event is independent of layer features, and it probably involves the adsorbed complex only (i.e., the Fe^3+^Phen bound to the exposed mineral surface). A third thermal event, with a maximum at about 550 °C, occurs in both samples between 400 and 700 °C with an almost identical mass loss (13.1 and 12.9 wt % in Kt-Fe^3+^Phen and Kt-FePhen-HPT-60d, respectively). The evolved gas analyses (Figure 3) suggest that it is related to the dehydroxylation of the octahedral sheet (emission of H_2_O, *m*/*z* = 18) and, simultaneously, to the thermal decomposition of the complex, with emission of H_2_O (*m*/*z* = 18), NO (*m*/*z* = 30), and CO_2_ (*m*/*z* = 44). The wide band observable for SO_2_ (*m*/*z* = 64) at about 500 °C in the not-exposed sample and barely visible in the exposed one (see magnification) can be ascribed to the thermal decomposition of the sulfate counterion of the complex.

### 3.3 Diffuse Reflectance (DR) UV–Vis–NIR Spectroscopy

**Figure 4:**
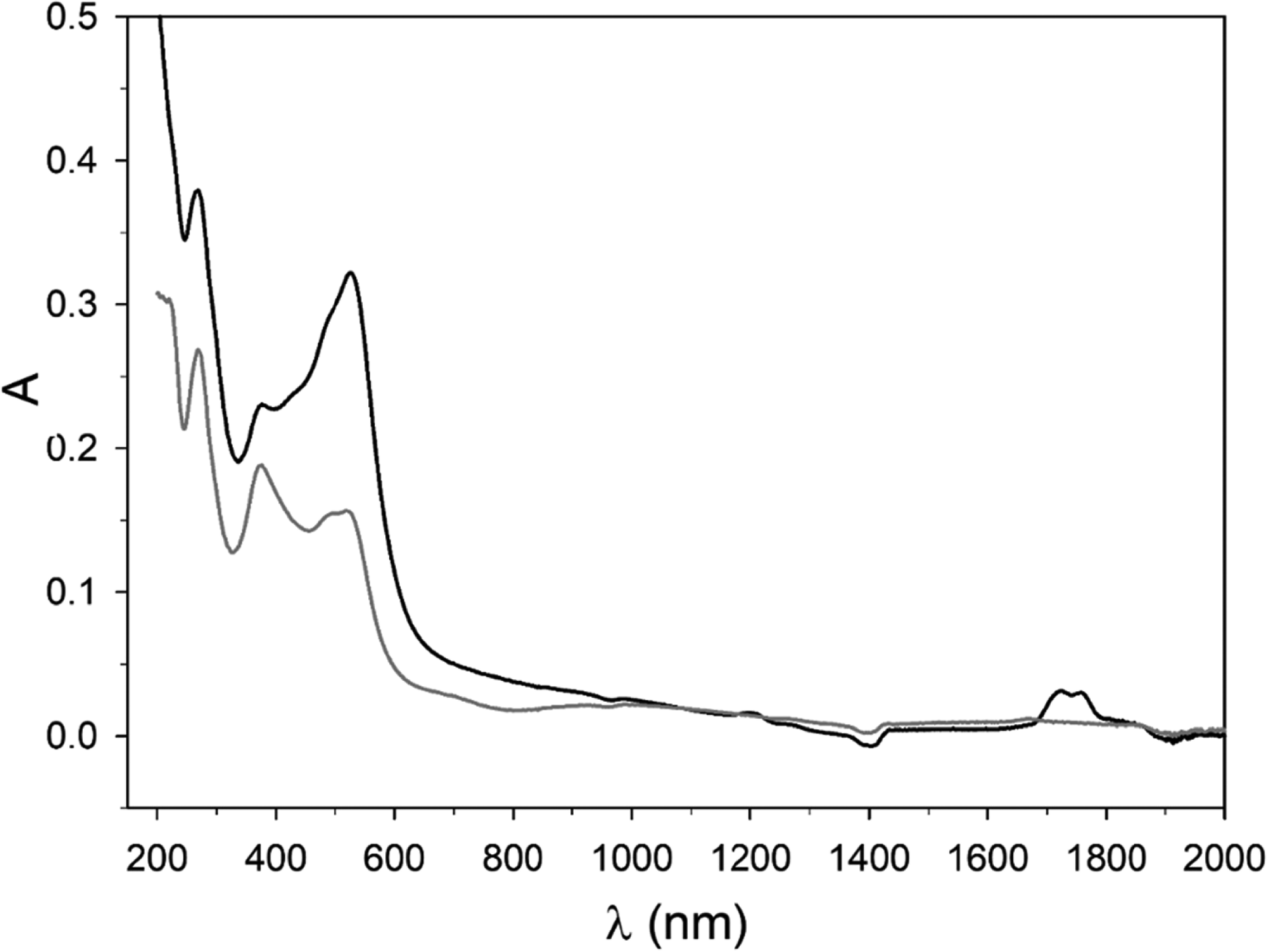
DR UV–Vis–NIR spectroscopy. Spectra of Kt-Fe^3+^Phen (gray line) and Kt-FePhen-HPT-60d (black line).

Figure 4 shows that in Kt-FePhen-HPT-60d the intensity of the absorption peak at 374 nm, which is related to the charge transfer band O^2–^ (bridge) → Fe^+3^ of the Fe^3+^Phen complex, is much lower than that in Kt-Fe^3+^Phen at the same wavelength. The intensity of the composite band at 526 nm related to the d → π* metal-to-ligand charge transfer (i.e., Fe^+2^ bound to the phenanthroline ligand)^40,51^ increases significantly in the spectrum of Kt-FePhen-HPT-60d compared to that of Kt-Fe^3+^Phen. In the latter, the presence of a very weak band indicates that iron is partially reduced during the immobilization of the complex on the kaolinite.

**Figure 5:**
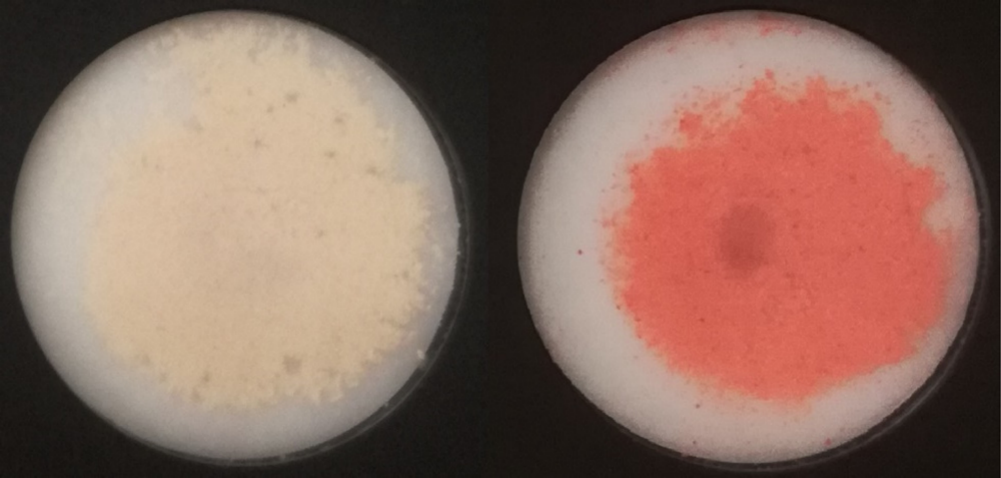
Change in color before and after HPT exposure. Samples Kt-Fe^3+^Phen (left) and Kt-FePhen-HPT-60d (right).

**Figure 6:**
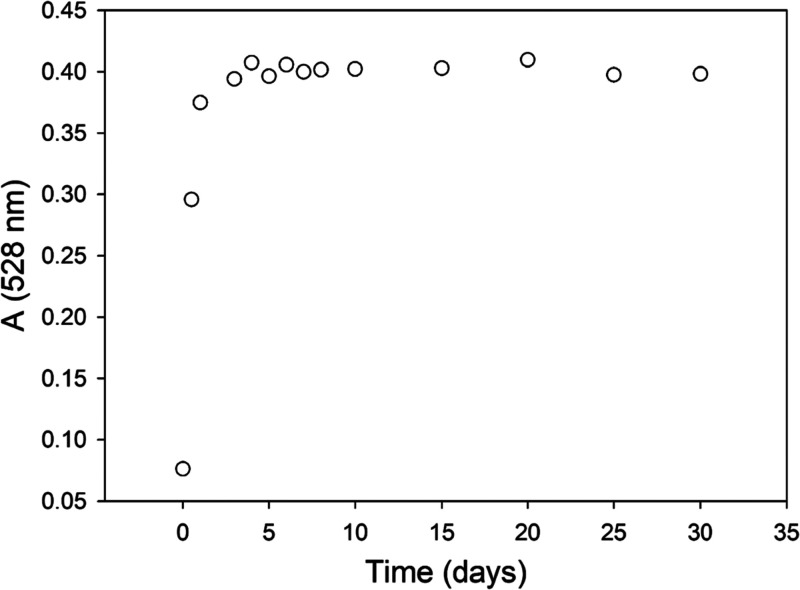
UV–Vis spectroscopy. Trend of intensity of the absorption signal at 528 nm as a function of exposure time to HPT. Standard deviation is inside the dimension of the symbol.

The corresponding change in color before and after exposure is clearly visible even to the naked eye as shown in Figure 5. Therefore, the UV–Vis region of the spectrum indicates that a significant fraction of trivalent iron in Kt-Fe^3+^Phen is reduced to divalent iron after HPT exposure, as evidenced in Figure 6, where the intensity of the peak at 528 nm is plotted versus time. As it will be detailed in the Section 4, this signal reaches a plateau after 5 days, in apparent contrast with the HPT adsorption kinetics which do not show a dwell (Figure 1). The strong peak at 254 nm can be confidently attributed to the π → π* transition of the Phen molecule in the Fe(III) complex.^36,52^ In the NIR zone of the exposed sample spectrum, three new signals appear at 1202 (weak), 1724, and 1758 nm. These peaks, not present in the Kt-Fe^3+^Phen spectrum, can be probably attributed to the formation of sulfurated species as a consequence of the Fe^+3^–thiol interaction.

### 3.4 IR Spectroscopy

**Figure 7:**
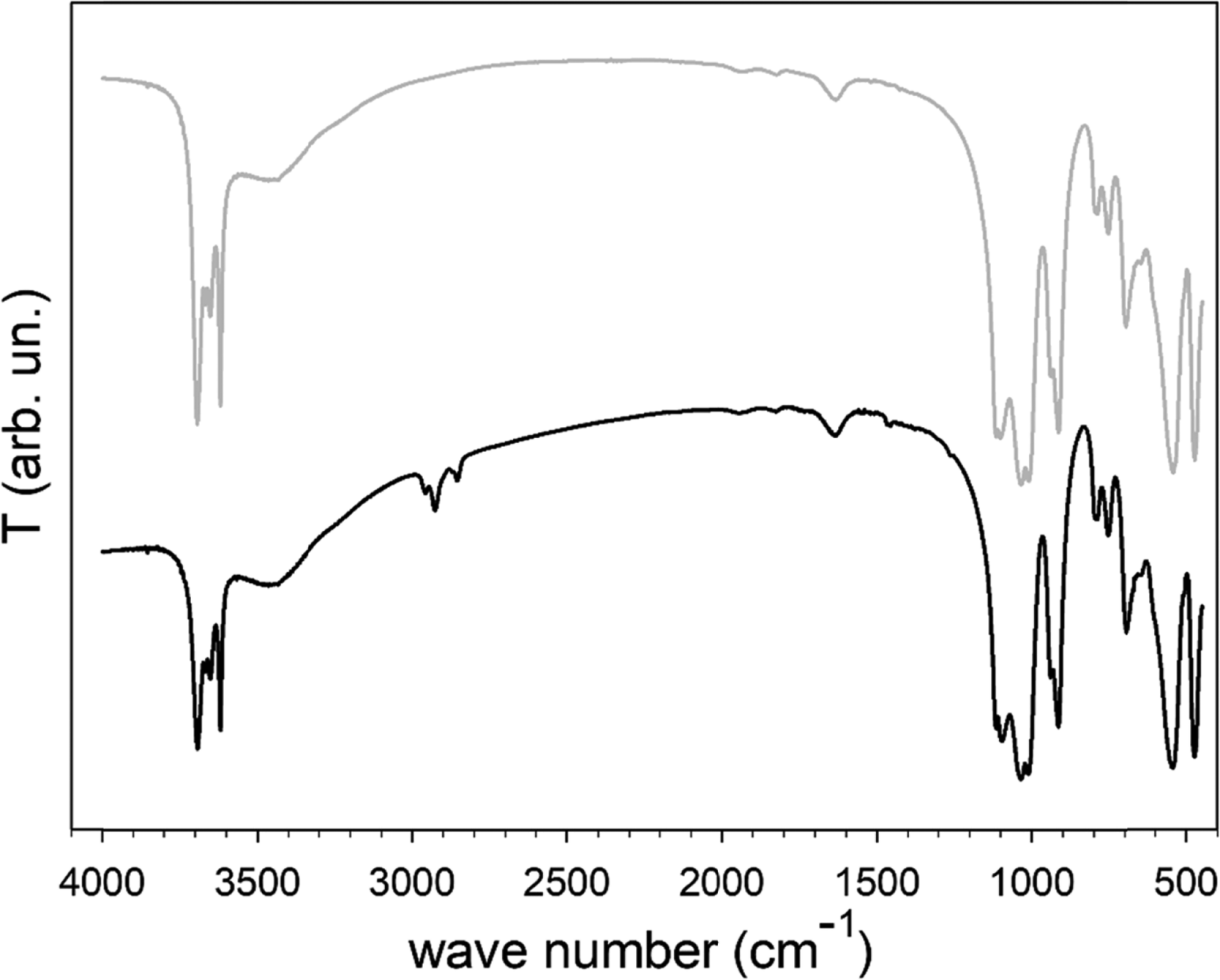
IR spectroscopy. Spectra of Kt-Fe^3+^Phen (gray line) and Kt-FePhen-HPT-60d (black line).

The IR spectra of Kt-Fe^3+^Phen and Kt-FePhen-HPT-60d are compared in Figure 7. In the exposed sample, the characteristic stretching signals of the aliphatic CH of the HPT chain are well evident at 2957, 2926, 2871 (weak), and 2856 cm^–1^. The signals at 2871 and 2856 cm^–1^ can be attributed to the symmetric stretching of CH_3_ and CH_2_ groups, respectively, while the two signals at 2957 and 2926 cm^–1^ are related to the asymmetric stretching of the CH_3_ and CH_2_ groups, respectively.^40^ In addition, in the exposed sample, new signals related to the HPT alkyl chain appear at 1466, 1455, 1378, and 1261 cm^–1^ and are related to the asymmetric bending of CH_3_, the symmetric bending of CH_2_ (both inside and alkyl chain and bound to a S atom, CH_2_–S), the symmetric bending (umbrella) of CH_3_, and the wagging of CH_2_ bound to a S atom, respectively.^53^ The stretching of the C–S bond, which should cause a weak absorption in the IR range of 600–800 cm^–1^,^54^ does not appear, and in this region, the two spectra are very similar. Likewise, neither the stretching of the S–H bond, which should occur between 2550 and 2600 cm^–1^, is observed suggesting that the interaction of HPT with iron involves the deprotonated form of HPT.

## 4 Discussion

Kaolinite is a good model to study the reaction mechanisms that mostly occur at the surface (i.e., on the edges of a finite sequence of tetrahedral/octahedral layers). In fact, the interlayer of kaolinite is remarkably less affected by the interactions with external molecules than, for example, that of montmorillonite. Actually, chemical analysis and TGA-MSEGA indicated that the Fe^+3^Phen complex is adsorbed on kaolinite; in addition, the simultaneous occurring of dehydroxylation and thermal decomposition of the complex suggests that hydrogen bonds are formed between the water molecules of Fe^+3^Phen and the exposed hydroxyl groups.

The major difference in the mode of interaction with HPT of hybrid kaolinite compared to montmorillonite is well evident for long interaction times. Like in montmorillonite, also in kaolinite the spectroscopic results clearly indicated that the reaction with HPT leads to the reduction of the Fe^3+^ in the complex to Fe^2+^, which is reasonably joined in a redox pathway to the oxidation of the trapped HPT to disulfide; however, while in montmorillonite HPT saturation is reached after about 2 weeks,^40^ this does not occur in kaolinite (Figure 1). As for montmorillonite,^40^ the formation of disulfide from the oxidation of thiol was checked by extraction with benzene of the reaction products obtained after 10 days of exposure to HPT. The resulting liquid phase after evaporation of benzene has been analyzed by Fourier transform infrared (FTIR) spectroscopy, and the resulting spectrum (Figure SI-1) is typical of disulfide.

The amount of HPT adsorbed on kaolinite after 60 days of exposure is 0.10940 moles per 100 g of Kt. Considering the amount of adsorbed complex (0.00114 moles per 100 g of Kt), it means a thiol/Fe^3+^Phen molar ratio of about 100:1, which is much higher than the 6:1 ratio found for the Fe^3+^Phen-functionalized montmorillonite,^40^ but also than those found for the functionalized sepiolites,^39^ where it varies from 40:1 to 65:1, mostly depending on the mineral structural ordering. This result is even more surprising considering the amount of complex respectively adsorbed by each clay mineral. In fact, previous thermodynamic studies on the adsorption properties of the Fe^+3^Phen showed a remarkable difference (about 40-fold) in the maximum number of moles of complex adsorbed by kaolinite (0.00114 moles per 100 g of Kt) and montmorillonite (0.0439 moles per 100 g of montmorillonite).^36^ The amount of Fe^+3^Phen adsorbed by sepiolite is much closer to that of kaolinite but, as mentioned, it is affected by mineral structural ordering and ranges between 0.0048 and 0.0152 moles per 100 g of sepiolite.^39^ This enhanced uptake of HPT by Kt-Fe^3+^Phen can be explained only by considering a continuous catalytic activity of Fe^3+^ toward the oxidation of thiol to disulfide according to the following reaction mechanisms (water molecules were omitted in the formula of the iron compounds for the sake of clarity):
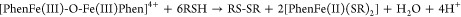
1
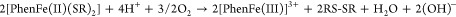
2
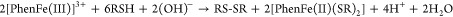
3or, alternatively:
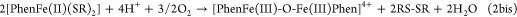

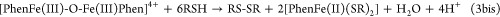


This reaction mechanism only in part overlaps those already hypothesized for hybrid montmorillonite and sepiolite. Nevertheless, the available data cannot allow to distinguish if the reaction proceeds through steps (2) and (3) or through steps (2bis) and (3bis), namely, if the reoxidized complex is mononuclear or dinuclear or else their mixture. Indeed, in the DR-UV–Vis spectrum of Kt-FePhen-HPT-60d the signal related to the charge transfer band O^2–^ (bridge) → Fe^+3^ of the Fe^3+^Phen complex is small but still present, thus accounting for the presence of the dinuclear complex in the pathway.

The charge of the free protons, which could be provided by the exposed edges of the kaolinite, compensates the decrease of the positive charge of the iron that, in consequence of the redox reaction, loses one positive charge. This mechanism also explains why the relatively fast reduction of trivalent iron shown in Figure 6 does not significantly affect the continuous uptake of HPT, allowing the hybrid kaolinite to outperform the HCM prepared with montmorillonite and sepiolite. A possible explanation of the different trapping behavior of kaolinite with respect to montmorillonite and sepiolite could be that the Fe^3+^Phen complex in kaolinite is adsorbed on the exposed surface as indicated by the absence of structural changes;^36^ in contrast, in montmorillonite and sepiolite, the complex is mostly intercalated between layers or confined in the zeolitic channels.^36,39^ In the former structural allocation, the complex can react more efficiently with atmospheric oxygen which is necessary to allow the discussed catalytic reaction. In addition, the progressive accumulation of reaction products within the montmorillonite interlayer (or zeolitic channels in sepiolite) probably makes it more difficult for oxygen to enter and diffuse. In contrast, the not-confined adsorption of the complex in kaolinite prevents its insulation and the consequent slowdown of the gas-trapping reaction. However, it cannot be excluded that the increase in hydrophobicity because of the accumulation of the disulfide delays the formation of products showing an ionic nature, see step (2).

In the IR spectra of hybrid kaolinite, also after 2 months of exposure to HPT vapors, the signal related to S–H stretching (2550–2600 cm^–1^, Figure 7) is lacking, confirming that the immobilization occurs through interaction of a thiolate form with the iron complex, excluding the presence of residual physio-adsorbed HPT on the surface, as already observed for functionalized montmorillonite.^40^ Unfortunately, the bands typical of the disulfide (w 705–570 cm^–1^ for C–S stretching and w 620–600 cm^–1^ for S–S stretching) fall on the strong and wide bands of the silicates and cannot be observed. Nevertheless, the presence of only one major species of sulfur derivatives (disulfide) is indirectly confirmed by TGA-MSEGA results (Figures 2 and 3) which showed that HPT is thermally released in a single thermal event, as well marked by the narrow and symmetrically shaped DTG signal with a maximum at 220 °C. In contrast, in montmorillonite two well-defined temperature ranges (i.e., 85–195 °C and 195–260 °C) were observed in which HPT is released as intact and fragmented molecules, respectively.^40^

## 5 Conclusions

The experimental data discussed above indicate that Kt-Fe^3+^Phen works with a different and remarkably better performant reaction mechanism to capture the volatile thiol than the Fe^3+^Phen-functionalized montmorillonite and sepiolite. The improvement is due to the occurring of a catalytic reaction with oxygen, facilitated by the location of the complex on the external surface, which restores the starting complex and allows an ongoing trapping activity.

Although a redox mechanism is always involved at the beginning of the reaction, in montmorillonite the trapping ends after 2 weeks of exposure, when the functionalized material reaches the saturation. This suggests that confining the Fe^3+^Phen complex between the layers may dramatically affect its long-term trapping ability. Therefore, while it is established that only the presence of Fe^3+^Phen may grant the uptake, once again it emerges that the type of layer plays a key role in controlling the development of the reaction as enhanced also by functionalized sepiolite. This result found for the 1:1 layered silicate kaolinite undoubtedly represents an exception, because the 2:1 layered silicates have traditionally been preferred for the preparation of organic–inorganic HCMs.

## Supporting Information Available

(i) A detailed description of the employed instrument and of the adopted experimental conditions and (ii) a picture (Figure SI-1) with the FTIR spectra of HPT and the reaction products (PDF)
