# Supplementary material for: Gaseous Heptanethiol Removal by a Fe3+-Phenanthroline–Kaolinite Hybrid Material
Source: ACS Omega. 2021 Nov 23;6(48):32589–96. doi: 10.1021/acsomega.1c04145 (PMC8655764; doi:10.1021/acsomega.1c04145)
Supplement: Supplementary file 1 — ao1c04145_si_001.pdf [file ao1c04145_si_001.pdf]

Gaseous heptanethiol removal by Fe^{3+}-phenanthroline-kaolinite hybrid material

Fabrizio Bernini, Elena Castellini, Maria Franca Brigatti, Beatrice Bighi, Marco Borsari, Daniele Malferrari*

Department of Chemical and Geological Sciences, University of Modena and Reggio Emilia, Via Campi 103, I-41125 Modena, Italy

* Corresponding author

SUPPLEMENTARY MATERIAL

Details on instruments and experimental conditions

UV-Vis and diffuse-reflectance (DR) UV-Vis measurements were performed using a UV-Vis spectrophotometer V-570 Jasco Instrument. The spectrophotometer was equipped with an integrating sphere attachment (Jasco model ISN-470) and the measurements were made in the range λ=220-900 nm using pristine kaolinite as reference.

FT-IR measurements were performed using a JASCO FT/IR 4700 (resolution: 0.4cm^{-1}). The samples were disks of KBr containing the kaolinite nanocomposites (typically 2% w/w) before and after exposure to heptanthiol.

The elemental analyses (C, N, S) were performed by a Carlo Erba Elemental Analyzer (Model 1106) on about 2 mg of material.

Thermogravimetric analyses (TGA) and *evolved gasses mass spectrometry* (MSEGA) were obtained using a Seiko SSC 5200 thermal analyzer coupled with a quadrupole mass spectrometer (ESS, GeneSysQuadstar 422) which allowed the analyses of the gases evolved during the thermal reactions. Gas sampling by the spectrometer is done at the outlet of the furnace chamber of the thermal analyzer via an inert, fused silicon capillary system, heated to prevent gases condensing. Gas analyses were carried out in Multiple Ion Detection mode (MID) to determine the nature of the evolved chemical species with temperature (or time). Background subtraction was used to obtain the point zero conditions before starting the MID analysis.
Measurements were performed on air-dried sample at the following experimental conditions: heating rate: 20°C/min; heating range: 25-1150°C; data measurement: every 0.5 sec; purging gas: ultrapure

helium, flow rate: 100μL/min. MID analyses was targeted to detect the m/z ratios17 and 18 for H_2O, 28 and 44 for CO_2, 30 for NO and NO_2, 34 for H_2S, 46 for NO_2, and 48, 64, 66 for SO_2, 55, 56, 70, 97, 98, and 132 for heptanethiol (where *m/z* is the dimensionless ratio between the mass number and the charge of an ion); SEM and FARADAY detector at 900V were employed with 1 sec of integration time on each measured mass with the exception of m/z=18 and 64 where 0.1 second were applied to avoid detector oversaturation. To limit differences in relative humidity, samples were equilibrated for 15 min inside the oven using a 100 μL/min flow of ultrapure helium before starting each measurement.

Supplementary figure

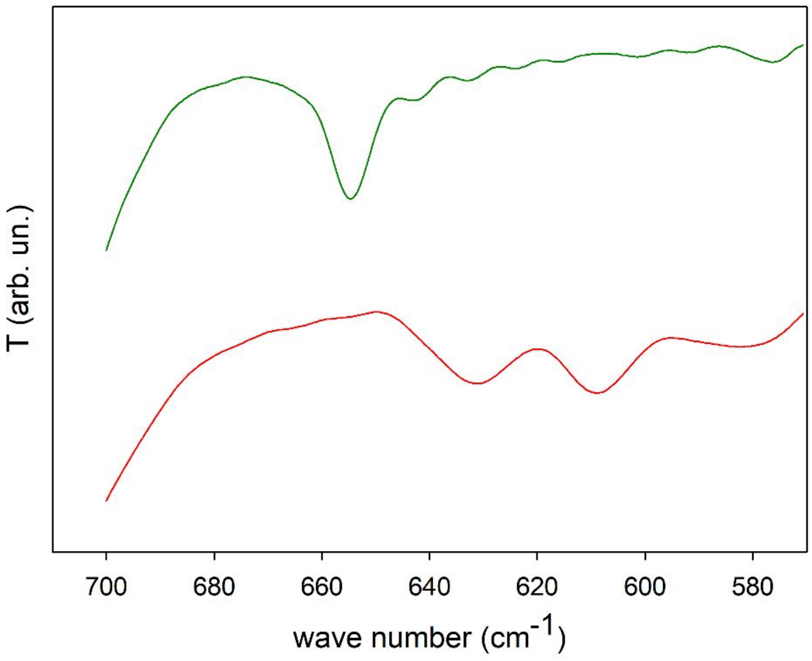

Figure SI-1. Detail of the FTIR spectra of heptanethiol (green) and the reaction product (red). In the spectrum of heptanethiol the band at 655nm is due to the C-S stretch, in the reaction product the bands at 632 nm and 606 nm agree with C-S and S-S stretch of a disulphide.[1]

REFERENCE

(1) Coates, J. Interpretation of Infrared Spectra, A Practical Approach. In *Encyclopedia of Analytical Chemistry*; Meyers, R. A., Ed.; John Wiley & Sons, Ltd: Chichester, UK, 2006; p a5606. https://doi.org/10.1002/9780470027318.a5606.
